# Associations of the Inflammatory Marker YKL-40 with Measures of Obesity and Dyslipidaemia in Individuals at High Risk of Type 2 Diabetes

**DOI:** 10.1371/journal.pone.0133672

**Published:** 2015-07-21

**Authors:** Stine B. Thomsen, Anette P. Gjesing, Camilla N. Rathcke, Claus T. Ekstrøm, Hans Eiberg, Torben Hansen, Oluf Pedersen, Henrik Vestergaard

**Affiliations:** 1 Section of Metabolic Genetics, The Novo Nordisk Foundation Center for Basic Metabolic Research, Faculty of Health and Medical Sciences, University of Copenhagen, Copenhagen, Denmark; 2 Center of Endocrinology and Metabolism, Department of Medicine, Copenhagen University Hospital, Herlev, Denmark; 3 Biostatistics, Department of Public Health, Faculty of Health and Medical Sciences, University of Copenhagen, Copenhagen, Denmark; 4 Department of Cellular and Molecular Medicine, Faculty of Health and Medical Sciences, University of Copenhagen, Copenhagen, Denmark; 5 Section of Molecular Diabetes & Metabolism, Institute of Clinical Research & Institute of Molecular Medicine, Faculty of Health Sciences, University of Southern Denmark, Odense, Denmark; 6 Institute of Biomedical Science, Faculty of Health and Medical Sciences, University of Copenhagen, Copenhagen, Denmark; 7 Faculty of Health Sciences, University of Aarhus, Aarhus, Denmark; Sapienza University of Rome, ITALY

## Abstract

**Introduction:**

Circulating levels of the inflammatory marker YKL-40 are elevated in cardiovascular disease and obesity-related type 2 diabetes (T2D), and serum YKL-40 levels are related to elements of dyslipidaemia.

**Objective:**

We aimed to investigate the associations between serum YKL-40 and obesity-related traits in a Danish sample of non-diabetic relatives to T2D patients and, furthermore, to estimate the heritability of YKL-40.

**Research Design and Methods:**

324 non-diabetic individuals with family relation to a T2D patient were included in the study. The participants underwent oral- and intravenous glucose tolerance tests for estimation of glucose tolerance and surrogate measures of insulin sensitivity. Anthropometric measures were retrieved and biochemical measures of the plasma lipid profile and serum YKL-40 levels were obtained. Association-analyses between serum YKL-40 and obesity-related traits and estimates of the narrow sense heritability of YKL-40 were based on a polygenic variance component model.

**Results:**

Fasting serum levels of YKL-40 were positively associated with waist-hip-ratio (p<0.001) and fasting plasma triglyceride levels (p<0.001). None of the insulin sensitivity indexes were significantly associated with YKL-40. According to the AE model, the familiality-estimate *h* ^2^ of YKL-40 was 0.45 (SE 0.13). When the ACE-model was applied, the heritability-estimate *h* ^2^ of YKL-40 did not reach statistical significance.

**Conclusions:**

Our results suggest a role of serum YKL-40 in obesity-related low grade inflammation, but do not indicate that YKL-40 is directly involved in the development of T2D.

## Introduction

Development of obesity-related diseases is thought to be connected to the initiation and establishment of a low grade inflammatory state characterized by elevated circulating levels of several inflammatory acute phase reactants, cytokines and cell adhesion molecules [1–3]. Among these is the glycoprotein YKL-40, also known as chitinase-3-like-1 (*CHI3L1*) [4]. YKL-40 is a 40 kDa heparin-and chitin-binding glycoprotein. It is secreted *in vitro* by several cell-types with relation to the innate immune system, e.g. activated macrophages and vascular smooth muscle cells [5;6]. Even though a receptor has yet to be discovered, it is generally accepted that YKL-40 plays an important part in the activation of the innate immune system and the remodeling of extracellular matrix [5;6]. Furthermore, YKL-40 is a potent angiogenic factor [7] and is thought to facilitate the formation of atherosclerotic plaques [8]. Finally, elevated serum YKL-40 levels have been found in cardiovascular disease [9;10], type 1 diabetes and type 2 diabetes (T2D) [11;12], and several types of cancer [13].

Previously, circulating YKL-40 levels were found to correlate positively with plasma levels of FFA and triglycerides in patients with T2D [11] and with levels of triglycerides in patients with stable coronary artery disease [14]. Similarly, in a Danish population-based study a positive association between levels of serum YKL-40 and plasma triglyceride have been reported [15]. Thus, we have previously proposed a role of YKL-40 in the development of dyslipidaemia [15], and we speculate that YKL-40 due to its angiogenic properties could be a factor involved in the expansion of the adipose tissue and thereby in the development of obesity.

The development of T2D is linked to obesity and the accumulation of ectopic fat [16]. Patients with T2D have higher circulating levels of several inflammatory markers than non-diabetic individuals [17] and it is known that the development of low grade inflammation precedes that of insulin resistance and T2D by many years [18]. Therefore, the aim of the present study was to evaluate the associations between fasting serumYKL-40 levels and obesity-related traits in a population of non-diabetic family members of T2D patients. Furthermore, we aimed to explore the heritability of serum YKL-40. Such insights might add to the current knowledge of the regulatory mechanisms behind the development of subclinical low-grade inflammation preceding T2D and cardiovascular disease.

## Materials and Methods

### Participants

The characteristics of the study participants have been reported previously [19]. Ninety-five families, each consisting of one patient (the proband) with verified T2D [20;21] and a varying number of family members without known diabetes were identified through the outpatient clinic at Steno Diabetes Center, Gentofte, Denmark and through an on-going study at the University of Copenhagen. All probands had diabetes onset after the age of 40 years and had no known history of type 1 diabetes. In this study, only the non-diabetic relatives were included. Thus, 337 individuals were eligible for inclusion, and serum samples of YKL-40 were available from 327 of these participants and were included in the final study sample. All individuals underwent a 4 hour oral glucose tolerance test (OGTT). 275 individuals had normal glucose tolerance and 52 had impaired glucose tolerance. Furthermore, 292 of the study participants underwent an intravenous glucose tolerance test (IVGTT).

### Anthropometric and biochemical measures

Measures of height, weight, abdominal and hip circumference were retrieved as reported previously [19;22]. Body mass index (weight (kg)/height (m^2^)) and waist-hip-ratio (WHR, waist circumference/hip circumference) was calculated. The fat percentage was measured by bio-impedance (Biodynamics BIA 310e, H.A.W consulting, Denmark).

Venous blood samples were drawn following a 12 hour overnight fasting. Serum YKL-40 was measured using an enzyme linked immune-ad-sorbent assay (ELISA) method (Quidel, USA). Measuring range was 20–300 ng/ml with intra-and interassay coefficients of variation of 0.058 and 0.06 respectively. Plasma glucose was analysed with a glucose oxidation technique (Granutest, Merch, Darmstadt, Germany). Serum insulin was analysed with an ELISA method with a narrow specificity excluding des (31,32 proinsulin split products)- and intact pro-insulin [23]. Plasma lipids were measured using standard laboratory techniques. Fasting samples of plasma glucose and serum insulin were analysed in triplicates.

### OGTT

All participants (non-diabetic) underwent a standard 75 g oral glucose tolerance test (OGTT) with venous blood sampled frequently at 10, 20, 30, 40, 50, 60, 75, 90, 105, 120, 140, 160, 180, 210 and 240 minutes.

### IVGTT

Within a week after the OGTT examination, 292 individuals underwent a tolbutamide modified, frequently sampled IVGTT after a 12 hour fast, as previously described [24]. Blood samples were retrieved at 33 time points during the 3 hours of the test. Glucose and tolbutamide were injected at t = 0 and t = 20 min, respectively.

### Ethics statement

Informed written consent was obtained from all participants prior to inclusion. The study was approved by the Local Ethics Committee of Copenhagen County (KA93033) and was in accordance with the principles of the Declaration of Helsinki II.

### Additional calculations

The homeostatic model assessment of insulin resistance (HOMA-IR) index was calculated as: (fasting plasma glucose ∙ fasting serum insulin)/22.5; and the OGTT-derived Matsuda index was calculated as previously described [25].

The OGTT-derived estimate of S_i_, BIGTT-S_i_, was calculated with insulin and glucose measurements at 0, 30 and 120 minutes [26].

The IVGTT-derived insulin sensitivity index (S_i_) was calculated using the Bergman MINMOD computer program as described previously [27].

### Statistical analyses

Statistical analyses were performed using the statistical software SOLAR [28] in order to account for the family-relation between participants. If residuals had a non-Gaussian distribution, they were log- transformed prior to analysis (BMI, triglyceride, cholesterol, HOMA-IR, S_i_ and the Matsuda index). When traits remained to show some degree of kurtosis, the t-distribution was used to reduce the effects of outliers. All baseline characteristics were analysed and presented according to YKL-40 quartile. Continuous variables were presented as mean (standard deviation (SD)) and categorical variables were presented as numbers. Analysis of variance (ANOVA) was used to compare the mean levels of baseline characteristics among the four groups of patients defined by the YKL-40 quartiles. Association-analyses and estimation of the narrow sense heritability of serum YKL-40 were based on a polygenic variance component model. Association-analyses between continuous values of YKL-40 as the explanatory variable and obesity-related traits were performed with adjustments for age and sex, or age, sex and BMI as stated in the table legend. Association-analyses between (log)YKL-40 and age were performed with age as the explanatory variable (adjusted for sex). Results were reported as β-coefficients (standard error (SE)) and p-values. Heritability estimates were calculated in two ways; i) from the AE-model which accounts for the additive genetic effect (A) and the unique environmental effect (E), and ii) from the ACE model, where the effect of the shared or common environment (C), i.e. the ‘household effect’, is also included. In the first model, the heritability-estimate is also termed familiality. The heritabilities are expressed as an estimated percentage of the total variance of YKL-40 between individuals and are reported as *h* ^2^ (SE). In the ACE model, the effect of the shared environment is reported as *c* ^*2*^ (SE), and the effect of the unique environment is reported as *e* ^*2*^.

## Results

Fasting serum samples of YKL-40 were available for 327 non-diabetic family members. Three serum YKL-40 outliers of 2484, 744 and 624 μg/L, respectively, were removed prior to analyses, yielding a total of 324 participants. It seemed reasonable to remove these three samples, since the YKL-40 values of the 25^th^ and 75^th^ percentile were 40 and 70 μg/l, respectively, thus leading us to suspect error of analyses in the three aforementioned cases. The vast majority of participants within each family were first degree relatives, i.e. parent-offspring or siblings. The nature of the relationships between participants within each family is displayed in Table 1.

**Table 1:** Number and type of relationship between pairwise comparisons of individuals within each family (from a total of 324 non-diabetic individuals).

N	Type of relationship
149	Parent-offspring
433	Siblings
3	Grandparent-grandchild
51	Avuncular
18	Half siblings
87	3^rd^ degree
72	4^th^ degree
5	Unrelated

Characteristics according to serum YKL-40 quartiles are displayed in Table 2. Mean age, BMI, plasma triglycerides, total plasma cholesterol and HOMA-IR showed an increase throughout the YKL-40 quartiles (p = 0.002, p = 0.002, p<0.0001, p<0.0001 and p = 0.009, respectively) whereas BIGTT- S_i_ decreased (p = 0.01). WHR showed an increase from the 2^nd^ through the 4^th^ quartile (p = 0.001) and the mean fat percentage was lower in the 1^st^ quartile, compared to quartiles 2–4 (p = 0.04). Levels of HDL, S_i_ and Matsuda indexes did not change significantly.

**Table 2:** Characteristics at baseline according to YKL-40 quartiles.

	1st quartile	2nd quartile	3rd quartile	4th quartile	p-value
YKL-40 range, μg/l	≤39	>39≤51	>51≤69	>69	
N	82	81	80	81	
Males, n (%)	42 (51)	25 (31)	30 (38)	38 (47)	
Age, years	39.2 (9.7)	40.1 (10.4)	45.6 (14.4)	52.1 (14.4)	**0.002**
**Measures of obesity**					
BMI, kg/m^2^	25.2 (4.4)	26.1 (4.9)	27.2 (4.8)	27.6 (5.2)	**0.002**
WHR	0.85 (0.09)	0.84 (0.09)	0.86 (0.09)	0.89 (0.10)	**0.001**
fat percentage	29.8 (9.1)	34.7 (10.6)	35.1 (10.9)	33.4 (11.1)	**0.04**
**Lipids**					
Triglyceride, mmol/l	1.07 (0.61)	1.21 (0.85)	1.49 (1.06)	1.62 (0.87)	**<0.0001**
Cholesterol, mmol/l	4.95 (1.03)	5.07 (1.16)	5.31 (1.29)	5.86 (1.08)	**<0.0001**
HDL, mmol/l	1.22 (0.30)	1.27 (0.33)	1.28 (0.31)	1.32 (0.39)	0.09
**Insulin resistance and sensitivity indexes**					
HOMA-IR	8.55 (5.15)	9.75 (7.19)	10.25 (8.60)	12.20 (10.75)	**0.009**
BIGTT- S_i_	8.93 (3.48)	7.98 (3.31)	7.71 (3.86)	7.36 (4.01)	**0.01**
S_i_	11.26 (5.86)	10.37 (5.97)	9.86 (5.47)	9.53 (6.99)	0.09
Matsuda	10.76 (5.36)	10.05 (5.90)	10.08 (5.95)	9.06 (5.34)	0.1

ANOVA. Values presented as mean (SD) or number (% within quartile). Abbreviations: BIGTT- S_i_, estimate of S_i_; BMI, body mass index; FFA, free fatty acids; HDL, high density lipoprotein cholesterol; HOMA-IR, homeostasis assessment model of insulin resistance; SD, standard deviation; S_i_, insulin sensitivity index; WHR, waist-hip-ratio.

### Associations between continuous values of serum YKL-40 and obesity-related traits

Data are presented in Table 3. YKL-40 was positively associated with age, when adjusting for sex (β = 0.032 (SE 0.0036), p<0.0001). WHR showed a positive association with YKL-40, when adjusting for age and sex (β = 0.00047 (SE 0.00012), p<0.001), however, this was not the case for BMI or the fat percentage. Plasma triglyceride levels also showed a positive association with YKL-40, when adjusting for age, sex and BMI (β = 0.0028 (SE 0.00079), p<0.001. This association was sustained with adjustments for age, sex and WHR, however, the level of significance decreased β = (0.0021 (0.00086), p = 0.016. The remaining lipid-variables (total cholesterol and HDL) and indexes of insulin resistance and–sensitivity (HOMA-IR, BIGTT-S_i_, S_i_ and Matsuda) did not display significant associations with YKL-40.

**Table 3:** Associations between serum YKL-40 and measures of obesity-related traits. Total population of non-diabetic individuals with removal of 3 YKL-40 outliers (n = 324).

	β-values (SE)	p-values
^1^Age, years	0.032(SE 0.0036)	**<0.0001**
^**2**^ **Measures of obesity**		
^4^BMI kg/m^2^	0.00018 (0.00030)	0.56
WHR	0.00047 (0.00012)	**<0.001**
Fat percentage	0.012 (0.018)	0.52
^**3**^ **Lipids**		
^4^Triglyceride, mmol/l	0.0028 (0.00079)	**<0.001**
^4^Cholesterol, mmol/l	0.0018 (0.00034)	0.60
HDL, mmol/l	0.00054 (0.00049)	0.27
^**3**^ **Insulin resistance and sensitivity indexes**		
^4^HOMA-IR	0.0013 (0.00097)	0.17
BIGTT- S_i_	-0.0021 (0.0049)	0.66
^4^S_i_	-0.00027 (0.0013)	0.83
^4^Matsuda	-0.0014 (0.0093)	0.14

Variance component model. Abbreviations: BIGTT-S_i_, estimate of S_i_; BMI, body mass index; FFA, free fatty acids; HDL, high density lipoprotein cholesterol; HOMA-IR, homeostasis assessment model of insulin resistance; SD, standard deviation; S_i_, insulin sensitivity index; WHR, waist-hip-ratio.

^1^Age used as the explanatory variable, YKL-40 log-transformed, analyses adjusted for sex.

^2^Analyses adjusted for age and sex.

^3^Analyses adjusted for age, sex and BMI.

^4^log-transformed.

### Heritability-estimates of YKL-40

The familiality-estimate, *h* ^*2*^, of YKL-40 was 0.45 (SE 0.13), meaning that 45% of the total variance of YKL-40 between individuals are caused by genetic influence or by a shared environment within the families. This familiality was only slightly reduced by adjustment for WHR and triglyceride (*h* ^2^ = 0.42 (SE: 0.13)), indicating that the familiality of YKL-40 is not under the same control as WHR and triglyceride levels. However, when taking the common environment into account, i.e. the ACE-model, *h* ^*2*^ of YKL-40 was reduced to 0.21 (SE 0.25), p = 0.2 and the effect of the shared environment was estimated to be *c* ^2^ = 0.11 (SE 0.11), p = 0.1, indicating that a fraction of familiarity could be caused by a shared environment.

## Discussion

In this study of 324 non-diabetic relatives to T2D patients, we show that fasting serum YKL-40 levels are positively associated with measures of obesity and dyslipidaemia, i.e. WHR and fasting serum triglyceride levels. Previously, serum YKL-40 levels have been found to be elevated in morbidly obese patients with various co-morbidities compared to lean, healthy subjects [29], and in morbidly obese T2D patients when compared to morbidly obese but glucose tolerant individuals [30;31]. However, a positive association between serum YKL-40 and BMI has not previously been documented in multivariate adjusted analyses [11;15;30]. In the present study, we found that serumYKL-40 levels and WHR were positively associated, (β = 0.00047 (SE 0.00012), p<0.001, which is in accordance with another study where serumYKL-40 was strongly correlated to measures of waist, and WHR, but not to BMI [31]. We have no obvious interpretation of these associations, but previous studies have suggested that the visceral adipose tissue could be an important source of released YKL-40 in obesity [31], and since YKL-40 is known to regulate other inflammatory markers, e.g. increase the expression of monocyte chemoattractant protein-1 (MCP-1) in alveolar macrophages of patients with chronic obstructive pulmonary disease [32], it is possible that YKL-40 plays a role in orchestrating the inflammatory response originating from the visceral adipose tissue. We also demonstrated a positive association between circulating fasting levels of YKL-40 and triglycerides (β = 0.0028 (SE 0.00079), p<0.001), which is in accordance with previous studies [11;14;15;33]. The relation and possible causality between YKL-40, WHR and triglyceride is not explored. However, the familiality estimates of YKL-40 are not to a major extent influenced by adjustments for WHR and triglycerides, suggesting that the observed associations between these traits are not caused by a common influence from the shared environment or shared genetics. Future longitudinal studies in humans and experimental studies in rodents may add to gain insights into the order of events and relationships underlying our observations.

Many of the inflammatory cytokines derived from the adipose tissue are known to have a direct influence on cells from several organ systems, e.g. resulting in altered insulin secretion from the pancreatic beta cells [34] and attenuated insulin sensitivity in muscle-, liver-, and adipose tissue [35]. The elevated circulating YKL-40-levels in patients with T1D and T2D [11;12;29;30], and associations with insulin resistance [11;30] could suggest that YKL-40 is implicated in the development of T2D. However, we did not find associations between serum YKL-40 levels and indexes of insulin resistance and–sensitivity in this population of non-diabetic family members of T2D patients, indicating that YKL-40 does in fact not play a role in the development of insulin resistance and T2D. Previous studies documenting that circulating levels of YKL-40 increase with increasing levels of albuminuria in both T1D and T2D patients [12;36;37] further suggest that the YKL-40 levels in these patients are rather a reflection of the low grade inflammation and developing atherosclerosis.

The genetic influence on circulating YKL-40 levels has been evaluated in previous studies. Ober et al [38] reported a narrow sense heritability-estimate, *h* ^*2*^, of 0.51 (SE 0.1), similar to the *h* ^*2*^ estimate from the AE model reported by us (*h* ^*2*^ = 0.45 (SE 0.13)). The previous study did not estimate the effect of the shared environment, however, this study was conducted in the Hutteries who constitute a very homogenous population because of the communal lifestyle, e.g. eating habits and housing environment differ very little between individuals. Therefore, these analyses are probably not inflated due to shared households, even though this is not accounted for in the model. When we applied the ACE-model to our heritability-analyses of YKL-40, we found that the estimate of the additive genetic effect decreased, but with large SE-estimates (*h* ^*2*^ = 0.21 (SE 0.25), p = 0.2). This is probably caused by the small sample size, rather than a shared household playing a major role in this respect. Several studies have shown that variations of the YKL-40 encoding gene, *CHI3L1*, influence the circulating levels of YKL-40 [38–42], and functional variants have been identified [43;44]. Thus, we know of genetic factors influencing the level of circulating YKL-40 despite the low level of estimated heritability.

A limitation of this study, apart from the population size, is the lack of available information on the participants’ medical history, except for the fact that participants were non-diabetic. It is therefore difficult to say whether the analyses are confounded by e.g. anti-inflammatory drug treatment or lipid lowering agents.

In summary, we evaluated the possible associations of fasting serum YKL-40 levels with measures of obesity and fasting plasma lipid levels. We found that YKL-40 was positively associated with WHR and fasting plasma triglyceride levels, suggestive of a role of YKL-40 in obesity-related low grade inflammation. However, we failed to demonstrate any associations between YKL-40 and measures of insulin resistance or insulin sensitivity, indicating that YKL-40 is not directly involved in the key pathophysiological features of T2D. Furthermore, we estimated the familiality-coefficient, *h* ^*2*^, of the inflammatory marker YKL-40 in a study sample of non-diabetic individuals related to a T2D patient, and the results are in line with previous reports suggesting that circulating YKL-40 levels are partly under genetic control.
